# Spectral Quasi-Linearization Method for Non-Darcy Porous Medium with Convective Boundary Condition

**DOI:** 10.3390/e21090838

**Published:** 2019-08-26

**Authors:** R. A. Alharbey, Hiranmoy Mondal, Ramandeep Behl

**Affiliations:** 1 Department of Mathematics, King Abdulaziz University, P.O. Box 80203, Jeddah 21589, Saudi Arabia; 2 Department of Mathematics, Durgapur Institute of Advanced Technology and Management, Maulana Abul Kalam Azad University of Technology, West Bengal 713212, India

**Keywords:** micropolar fluid, boundary layer flow, quasi-linearization, Chebyshev spectral collocation method, entropy generation

## Abstract

The boundary layer micropolar fluid over a horizontal plate embedded in a non-Darcy porous medium is investigated in this study. This paper is solely focused on contributions oriented towards the application of micropolar fluid flow over a stretching sheet. The prime equations are renewed to ordinary differential equations with the assistance of similarity transformation; they are then subsequently solved numerically using the spectral quasi-linearization method (SQLM) for direct Taylor series expansions that can be applied to non-linear terms in order to linearize them. The spectral collocation approach is then applied to solve the resulting linearized system of equations. The paper acquires realistic numerical explanations for rapidly convergent solutions using the spectral quasi-linearization method. Convergence of the numerical solutions was monitored using the residual error of the PDEs. The validity of our model is established using error analysis. The impact of different geometric parameters on angular velocity, temperature, and entropy generation numbers are presented in graphs. The results show that the entropy generation number decelerates with an increase in Reynolds number and Brinkmann number. The velocity profile increases with the increasing material parameter. The results indicate that the fluid angular velocity decreases throughout the boundary layer for increasing values of the material parameter.

## 1. Introduction

Micropolar fluids are those in which the local micro-structure and intrinsic motion of fluid particles are considered in the flow regimen. Examples of micropolar fluids include industrial collodal fluids, polymeric suspensions, and liquid crystals. The theory of micropolar fluids, which was championed by Eringen [1,2], describes fluids that are composed of rigid and randomly oriented particles that are suspended in a viscous medium [3].

Non-Newtonian fluids, a class of fluids to which micropolar fluids belong, have many applications in engineering, agriculture, meteorology, industry, and so on. Examples include paints, colloidal fluids, ferro-liquids, polymeric fluids, exotic lubricants, and many others. The presence of dust in the air and blood flow in veins, arteries, and capillaries may also be studied using micropolar fluid dynamics. The usual momentum equations of fluid flow, commonly referred to as Navier–Stokes equations, are inadequate in fully describing flow of fluids at the nano and micro scale [4]. As a result of this limitation, the Navier–Stokes equations (also known as momentum equations) are used in conjunction with an additional model equation that accounts for angular momentum [5].

Increased interest in micropolar fluids has been demonstrated worldwide in recent years. Ishak et al. [6] studied dual solutions in the flow of micropolar fluids, while Poulomi et al. [7] analyzed dual solutions of the heat and mass transfer of a nanofluid over a stretching/shrinking sheet with thermal radiation. Kameswaran et al. [8] investigated dual solutions of a Casson fluid over, another example of a non-Newtonian fluid. Other examples of non-Newtonian fluids include a Sutterby fluid, Carreau-Yasuda fluid, visco-elastic fluid, psuedo-elastic fluid, Ellis fluid, and Williamson fluid, among others [9].

Tremendous amounts of time and effort have been invested by researchers in the study of non-Newtonian fluids in recent years. This can largely be attributed to the fact that most regularly used liquids, be it in the home or in engineering and technology, are to a great extent identifiable as being non-Newtonian [9].

Many researchers worldwide have focussed great attention on the concept of the boundary layer, which can be defined as a very thin layer close to the body or surface in which the viscosity of fluid particles is significant. Boundary layer theory has undoubtedly been responsible for modern advances in space flight, air travel, ship transport, technological warfare, motor sports, irrigation systems, biomedical technologies, etc, most of which have a role to play in the pleasures, comforts, and necessities of modern day life [10].

The phenomena of boundary layer flow over stretching/shrinking surfaces has extensive engineering applications, such as the cooling process of metallic plates in a cooling bath, the aerodynamic extrusion of plastic sheets, hot rolling, metal spinning, the condensation process of a boundary layer along liquid film, artificial fibers, glass-fiber production, paper production, and drawing of plastic films [11,12,13].

Pioneering studies on boundary layer flow over solid surfaces were done by Sakiadis [14] and followed up later by Liao [3,15], who studied flow over stretching permeable or impermeable walls and solved the system of equations by employing the homotopy analysis method, which is classified as an analytic method.

RamReddy et al. [16] studied mixed convection of a micropolar fluid over a permeable vertical plate with convective boundary condition and solved the system using spectral quasi-linearization method (SQLM). Their findings revealed that dual solutions exist for certain values of mixed convection parameter.

Most real-life phenomena, such as heat and mass transfer, fluid flow, and biological and engineering processes, are modeled by non-linear partial differential equations. By their nature, such equations are complex and very difficult to solve exactly [10,17,18]. It is for this reason that numerical methods, such as the spectral quasi-linearization, finite difference [17], and the Runge–Kutta method, have to be used to solve these and similar types of problems.

The works reported above and the references contained therein (as well as other works not reported here) have focussed on the heat transfer aspects of micropolar fluids embedded in a non-Darcian porous medium with a magnetic field.

Other numerical methods include the finite element method, the Runge–Kutta–Fehlberg method [19], the Keller-box method [20], and the shooting technique [21]. In this work, we apply the Chebyshev spectral quasi-linearization method to solve a problem involving the flow of a micropolar fluid, for the reason that spectral methods are renowned for their accuracy and precision [22].

## 2. Mathematical Formulations

**Figure 1:**
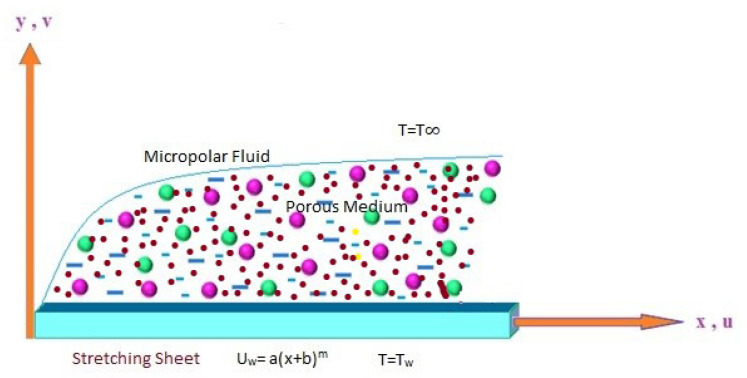
Schematic diagram of the problem under consideration.

In this study, a two-dimensional incompressible flow of a micropolar fluid is considered. The fluid flows steadily over a stretching/shrinking sheet that is impermeable and has the physical properties $u_{w}=a{\left(x+b\right)}^{m}$, $T_{w}=T_{\infty }+c{\left(x+b\right)}^{\lambda }$, where the parameters a, *b*, and *m* are related to the shrinking or stretching speed of the surface, while *c*, *b*, and λ are related to the temperature of the surface [5]. The *x*-axis lies in the direction parallel to the surface of the sheet and the *y*-axis is perpendicular to it (see Figure 1); *u* and *v* are the velocity components in the *x* and *y* directions, respectively.

The model equations emanate from the principles mass, linear momentum, angular momentum, and energy conservation and are given as (refer to [5])

(1) $$\begin{array}{c} \frac{\partial u}{\partial x}+\frac{\partial v}{\partial y}=0,\\ u\frac{\partial u}{\partial x}+v\frac{\partial u}{\partial y}=\left(\frac{\mu +k}{\rho }\right)\frac{\partial^{2}u}{\partial y^{2}}+\frac{k}{\rho }\frac{\partial N}{\partial y}-\frac{\sigma B_{0}^{2}}{\rho }u-\frac{\nu }{k}u-\frac{C_{b}}{\sqrt{k}}u^{2},\\ \rho j\left(u\frac{\partial N}{\partial x}+v\frac{\partial N}{\partial y}\right)=\frac{\partial }{\partial y}\left(\gamma \frac{\partial N}{\partial y}\right)-k\left(2N+\frac{\partial u}{\partial y}\right),\\ u\frac{\partial j}{\partial x}+v\frac{\partial j}{\partial y}=0,\\ u\frac{\partial T}{\partial x}+v\frac{\partial T}{\partial y}=\alpha \frac{\partial^{2}T}{\partial y^{2}}. \end{array}$$

The associated boundary conditions are as follows:

(2) $$\begin{array}{c} \begin{array}{c} u=u_{w},\;v=j=0,\;T=T_{w},\;N=-\frac{1}{2}\frac{\partial u}{\partial y}\;\;\mathrm{at}\;y=0,\\ u\to 0,\;N\to 0,\;T\to T_{\infty }\;\;\mathrm{as}\;y\to +\infty , \end{array} \end{array}$$

where *N* is the angular velocity (also termed the micro-rotation). Note that rotation is in the x−y plane. jandγ are the micro-inertia density and spin gradient of the fluid, respectively. The parameters μ,α, and *k* are the viscosity, the thermal conductivity, and the vortex viscosity of the fluid, respectively.

Let $\gamma =\mu (1+\frac{\Omega }{2})j$, where the material parameter $\Omega =\frac{k}{\mu }$ represents the dimensionless viscosity ratio [5]. We note here that Ω=0 points to the flow of a viscous and incompressible Newtonian fluid.

(3) $$\begin{array}{c} u\frac{\partial T}{\partial x}+v\frac{\partial T}{\partial y}=\alpha \frac{\partial^{2}T}{\partial y^{2}}. \end{array}$$

Let ψ denote the stream function such that $u=\frac{\partial \psi }{\partial y}$ and $v=-\frac{\partial \psi }{\partial x}$ and introduce the following dimensionless variables:

(4) $$\begin{array}{c} \xi =\sqrt{\frac{a(1+m)}{2\nu }}{\left(x+b\right)}^{\frac{m-1}{2}}y,\;\theta \left(\xi \right)=\frac{T-T_{\infty }}{c{\left(x+b\right)}^{\lambda }},\\ \psi =a\sqrt{\frac{2\nu }{a(1+m)}}{\left(x+b\right)}^{\frac{m-1}{2}}F\left(\xi \right),\;N=a\sqrt{\frac{a(1+m)}{2\nu }}{\left(x+b\right)}^{(3m-1)/2}h\left(\eta \right),\\ j=\frac{2\nu }{a(1+m)}{\left(x+b\right)}^{1-m}g\left(\xi \right), \end{array}$$

where ν denotes the kinematic viscosity of the fluid and a≠0 and a(m+1)>0.

Applying variables (4) in Equations (2)–(4) and Equation (3), the system becomes

(5) $$\begin{array}{c} \left(1+\Omega \right)F^{\prime \prime \prime }+FF^{\prime \prime }+\Omega h^{\prime }-\frac{2m}{1+m}{\left(F^{\prime }\right)}^{2}-MF^{\prime }-k_{1}F^{\prime }-F^{*}F^{\prime 2}=0,\\ \left(1+\frac{\Omega }{2}\right)\left(gh^{\prime \prime }+g^{\prime }h^{\prime }\right)+\left(Fh^{\prime }-\left(1+\frac{\Omega }{2}\right)F^{\prime }h\right)-\Omega \left(2h+F^{\prime \prime }\right)=0,\\ \left(1-m\right)F^{\prime }g-\frac{1+m}{2}Fg^{\prime }=0,\\ \theta^{\prime \prime }+Pr\left(F\theta^{\prime }-\frac{2\lambda }{1+m}F^{\prime }\theta \right)=0, \end{array}$$

where $M=\frac{\sigma B_{0}^{2}}{\rho a}{\left(x+b\right)}^{1-m}$ is the magnetic field parameter, $k_{1}=\frac{\nu {\left(x+b\right)}^{1-m}}{k}$ is the porous parameter, $F^{*}=\frac{C_{b}\left(x+b\right)}{\sqrt{k}}$ is the local inertia co-efficient, and $Pr=\frac{\nu }{\alpha }$ is the Prandtl number.

The boundary conditions for Equation (5) are

(6) $$\begin{array}{c} F\left(0\right)=0,\;\theta \left(0\right)=1,\;g\left(0\right)=0,\;h\left(0\right)=-\frac{1}{2}F^{\prime \prime }\left(0\right),\\ F^{\prime }\left(0\right)=1,\;\theta \left(\infty \right)=0,\;F^{\prime }\left(\infty \right)=0,\;h\left(\infty \right)=0. \end{array}$$

The above physical quantities of interest are $C_{f}$, $Nu_{x}$, $M_{w}$, which denote the local skin friction coefficient, the local Nusselt number, and the local couple stress at the surface, respectively. They are defined below as

(7) $$\begin{array}{c} C_{f}=\frac{\tau_{w}}{\frac{\rho u_{w}^{2}}{2}},\;Nu_{x}=\frac{\left(x+b\right)q_{w}}{k(T_{w}-T_{\infty })},\;M_{w}=\gamma {\left(\frac{\partial N}{\partial y}\right)}_{y=0}. \end{array}$$

Here, $\tau_{w}$ and $q_{w}$, which respectively represent the surface shear stress and the surface heat flux, are given by

(8) $$\begin{array}{c} \tau_{w}={\left[\left(\mu +k\right)\frac{\partial u}{\partial y}+kN\right]}_{y=0},\;q_{w}=-k{\left(\frac{\partial T}{\partial y}\right)}_{y=0}. \end{array}$$

Using the similarity variables in (4), we obtain

(9) $$\begin{array}{c} 0.5C_{f}Re_{x}^{0.5}=\pm \sqrt{0.5|1+m|}\left(1+0.5\Omega \right)F^{\prime \prime }\left(0\right) \end{array}$$

(10) $$\begin{array}{c} Nu_{x}Re_{x}^{0.5}=-\sqrt{0.5|1+m|}\theta^{\prime }\left(0\right), \end{array}$$

(11) $$\begin{array}{c} \;M/\mu u_{w}{\left(x\right)}^{m-1}=\left(1/2C\right)\left(1+m\right)\left(1+0.5\Omega \right)h^{\prime }\left(0\right), \end{array}$$

where $Re_{x}=\left|u_{w}\left(x+b\right)/\nu \right|$ gives the local Reynolds number.

## 3. Numerical Solution

Equation (5) forms a coupled system. We solve this system by firstly linearizing this pair of equations. We do this by utilizing the quasi-linearization technique and then by subsequently applying the Chebyshev spectral collocation method to solve the resulting system. A background theory of these procedures is given in the next subsection.

### 3.1. Quasi-Linearization

Consider a system of *n* non-linear differential equations which we write, without loss of generality, as

(12) $$\begin{array}{cc} \Gamma_{1}\left[H_{1},H_{2},\ldots ,H_{n}\right] & =0 \end{array}$$

(13) $$\begin{array}{cc} \Gamma_{2}\left[H_{1},H_{2},\ldots ,H_{n}\right] & =0\\ \vdots & = \end{array}$$

(14) $$\begin{array}{cc} \Gamma_{n}\left[H_{1},H_{2},\ldots ,H_{n}\right] & =0, \end{array}$$

where

(15) $$\begin{array}{cc} H_{1} & =\{f_{1}\left(\eta \right),f_{1}^{\prime }\left(\eta \right),f_{1}^{\prime \prime }\left(\eta \right),\ldots ,f_{1}^{\left(p\right)}\left(\eta \right)\} \end{array}$$

(16) $$\begin{array}{cc} H_{2} & =\{f_{2}\left(\eta \right),f_{2}^{\prime }\left(\eta \right),f_{2}^{\prime \prime }\left(\eta \right),\ldots ,f_{2}^{\left(p\right)}\left(\eta \right)\}\\ \vdots & = \end{array}$$

(17) $$\begin{array}{cc} H_{n} & =\{f_{n}\left(\eta \right),f_{n}^{\prime }\left(\eta \right),f_{n}^{\prime \prime }\left(\eta \right),\ldots ,f_{n}^{\left(p\right)}\left(\eta \right)\}. \end{array}$$

Here, *p* denotes the order of differentiation, while $f_{k}\left(\eta \right)$ and $\Gamma_{k}$ denote the solutions of the system and the non-linear operators containing all the spatial derivatives of $f_{k}\left(\eta \right)$ for k=1,2,…,n, respectively.

It is assumed that the solution can be approximated using the Lagrange interpolation polynomial of the form

(18) $$\begin{array}{c} f_{k}\left(\eta \right)=\sum_{j=0}^{N_{\eta }}f_{k}\left(\eta_{j}\right)L_{j}\left(\eta \right), \end{array}$$

for k=0,1,…,n where

(19) $$\begin{array}{c} L_{j}\left(\eta \right)=\prod_{\begin{array}{c} j=0\\ j\neq k \end{array}}^{N_{\eta }}\frac{\eta -\eta_{k}}{\eta_{j}-\eta_{k}} \end{array}$$

and

(20) $$\begin{array}{c} L_{j}\left(\eta \right)=\left\{\begin{array}{cc} 0, & \;\;j\neq k,\\ 1, & \;\;j=k. \end{array}\right. \end{array}$$

The grid points $\eta_{j}$ for j=0,1,…,n which are considered in this study are termed Chebyshev–Gauss–Lobatto grid points and are defined as

(21) $$\begin{array}{c} \left\{\eta_{j}\right\}=\cos\left(\frac{\pi j}{N_{\eta }}\right). \end{array}$$

The system of the *n*-non-linear differential equations under consideration is then linearized by using the quasi-linearization technique, which is outlined in great detail by Bellman and Kalaba [23].

Thus, applying the quasi-linearization technique leads one to obtain a coupled system *n* linear of ordinary differential equations given as follows:

(22) $$\begin{array}{cc} \sum_{s=0}^{p}a_{1,s,r}^{\left[1\right]}\left(\eta \right)f_{1,r+1}^{\left(s\right)}\left(\eta \right)+\sum_{s=0}^{p}a_{2,s,r}^{\left[1\right]}\left(\eta \right)f_{2,r+1}^{\left(s\right)}\left(\eta \right)+\ldots +\sum_{s=0}^{p}a_{n,s,r}^{\left[1\right]}\left(\eta \right)f_{n,r+1}^{\left(s\right)}\left(\eta \right) & =R_{1}\left(\eta \right) \end{array}$$

(23) $$\begin{array}{cc} \sum_{s=0}^{p}a_{1,s,r}^{\left[2\right]}\left(\eta \right)f_{1,r+1}^{\left(s\right)}\left(\eta \right)+\sum_{s=0}^{p}a_{2,s,r}^{\left[2\right]}\left(\eta \right)f_{2,r+1}^{\left(s\right)}\left(\eta \right)+\ldots +\sum_{s=0}^{p}a_{n,s,r}^{\left[2\right]}\left(\eta \right)f_{n,r+1}^{\left(s\right)}\left(\eta \right) & =R_{2}\left(\eta \right) \end{array}$$

(24) $$\begin{array}{cc} \vdots & =\\ \sum_{s=0}^{p}a_{1,s,r}^{\left[n\right]}\left(\eta \right)f_{1,r+1}^{\left(s\right)}\left(\eta \right)+\sum_{s=0}^{p}a_{2,s,r}^{\left[n\right]}\left(\eta \right)f_{2,r+1}^{\left(s\right)}\left(\eta \right)+\ldots +\sum_{s=0}^{p}a_{n,s,r}^{\left[n\right]}\left(\eta \right)f_{n,r+1}^{\left(s\right)}\left(\eta \right) & =R_{n}\left(\eta \right), \end{array}$$

where $a_{n,s,r}^{\left[k\right]}\left(\eta \right)=\frac{\partial \Gamma_{k}}{\partial f_{n,r}^{\left(s\right)}}$, where s=0,1,2,…,p are the variable coefficients of $f_{n,r+1}^{\left(s\right)}$ that correspond to the *k*th equation for k=1,2,…,n.

The right hand side of the *k*th equation is given by

(25) $$\begin{array}{c} R_{k}\left(\eta \right)=\sum_{s=0}^{p}a_{1,s,r}^{\left[k\right]}\left(\eta \right)f_{1,r}^{\left(s\right)}\left(\eta \right)+\sum_{s=0}^{p}a_{2,s,r}^{\left[k\right]}\left(\eta \right)f_{2,r}^{\left(s\right)}\left(\eta \right)+\ldots +\sum_{s=0}^{p}a_{n,s,r}^{\left[k\right]}\left(\eta \right)f_{n,r}^{\left(s\right)}\left(\eta \right)-\Gamma_{k}\left[H_{1,r},H_{2,r},\ldots ,H_{n,r}\right]. \end{array}$$

By following the above procedures, the linearized system of (5) becomes

(26) $$\begin{array}{c} \begin{array}{c} \left(1+\frac{\Omega }{2}\right)F^{\prime \prime \prime }+F_{r+1}F_{r+1}^{\prime \prime }-2\frac{2m}{1+m}F_{r}^{\prime }F_{r+1}^{\prime }+F_{r}^{\prime \prime }F_{r+1}=R_{1},\\ -\frac{2\lambda }{1+m}Pr\theta_{r}F_{r+1}^{\prime }+Pr\theta_{r}^{\prime }F_{r+1}+\theta_{r+1}^{\prime \prime }+PrF_{r}\theta_{r+1}^{\prime }=R_{2}, \end{array} \end{array}$$

where $R_{1}=-\frac{2m}{1+m}{\left(F_{r}^{\prime }\right)}^{2}+Fr^{\prime \prime }Fr$ and $R_{2}=-\frac{2\lambda }{1+m}Pr\theta_{r}F_{r}^{\prime }+Pr\theta_{r}^{\prime }F_{r}$, and the associated boundary conditions are

(27) $$F_{r+1}\left(0\right)=0,\;\theta_{r+1}\left(0\right)=1,F_{r+1}^{\prime }\left(0\right)=1,\;\theta_{r+1}\left(\infty \right)=0,\;F_{r+1}^{\prime }\left(\infty \right)=0.$$

### 3.2. Spectral Collocation

We solve the linearized systems (22) to (24) by evaluating at Chebyshev–Gauss–Lobatto grid points $\eta_{i}$, for $i=0,1,\ldots ,N_{\eta }$.

The values of the derivatives at the grid points are defined as

(28) $$\begin{array}{c} \frac{df_{n}}{d\eta }{|}_{\left(\eta_{i}\right)}=\sum_{\omega =0}^{N_{\eta }}D_{i\omega }f_{n}\left(\eta_{\omega }\right), \end{array}$$

where $D_{i\omega }f_{n}\left(\eta_{\omega }\right)=\frac{dL_{\omega }\left(\eta_{j}\right)}{d\eta }.$

Higher order derivatives are defined as

(29) $$\begin{array}{c} \frac{d^{p}f_{n}}{d\eta^{p}}{|}_{\left(\eta_{i}\right)}=D^{p}F_{n}, \end{array}$$

where $D^{p}F_{n}=\sum_{\omega =0}^{N_{\eta }}D_{i\omega }^{p}f_{n}\left(\eta_{\omega }\right)$ and $F_{n}={\left[f_{n}\left(\eta_{0}\right),f_{n}\left(\eta_{1}\right),\ldots ,f_{n}\left(\eta_{N_{\eta }}\right)\right]}^{T}$, and *T* denotes the transpose of the matrix.

It must be noted that before we apply Chebyshev spectral collocation to our linearized system of equations, we must change the domain of the problem from η=[a,b] to z=[−1,1] by using the transformation $\eta =a+\frac{b-a}{2}\left(z+1\right)$, where a=0 and *b* represents the value of η at infinity. For the purpose of our study, we used b=10 for convenience.

As a result of the above transformation, we obtain the transformed Chebyshev derivative matrices as follows:

(30) $$\begin{array}{ccc} {\left(D1\right)}_{i\omega } & = & 2*D_{i\omega }, \end{array}$$

(31) $$\begin{array}{ccc} {\left(D2\right)}_{i\omega } & = & {\left(D1\right)}_{i\omega }*D1_{i\omega }, \end{array}$$

(32) $$\begin{array}{ccc} {\left(D3\right)}_{i\omega } & = & {\left(D2\right)}_{i\omega }*D1_{i\omega }. \end{array}$$

We select the initial guesses so that they satisfy the boundary conditions of our system. We chose the initial guesses for our system of two equations as $F_{0}\left(\eta \right)=1-e^{-\eta }$ and $\theta_{0}=e^{-\eta )}$, and $h_{0}\left(\eta \right)=-\frac{1}{2}F^{\prime \prime }\left(0\right)e^{-\eta }$. The system is initially solved by the spectral quasi-linearization method and then the solution for h(η) is deduced from the solution for *F*.

For a more detailed treatment of spectral methods, we refer you to Canuto et al. [24], Trefethen [25], RamReddy [16], and Motsa et al. [26].

## 4. Entropy Generation Analysis

Entropy generation is dependent on the reversibility of a specified procedure. In an isolated system, entropy tends to increase with time, but remains steady for reversible reactions. As a result of the increasing application of nanofluids and nanoparticles in engineering and medical applications, it is imperative to investigate and study the impact of these nanoparticles on entropy generation in real life. This study focused on entropy generation along the sheet of magneto-micropolar nanofluids.

The volumetric rate of local entropy generation $S_{gen}^{\prime \prime \prime }$ for two-dimensional flow is given below:

(33) $$\begin{array}{c} S_{gen}^{\prime \prime \prime }=\underset{S_{th}}{\underset{︸}{\frac{k_{f}}{T_{\infty }^{2}}{\left(\frac{\partial T}{\partial y}\right)}^{2}}}+\underset{S_{dis}}{\underset{︸}{\frac{\left(\mu +K\right)}{T_{\infty }}{\left(\frac{\partial u}{\partial y}\right)}^{2}}}+\underset{S_{rot}}{\underset{︸}{\frac{\gamma }{T_{\infty }}{\left(\frac{\partial N}{\partial y}\right)}^{2}}}. \end{array}$$

Equation (33) reveals that the entropy generation is a contribution of six sources. The first source is caused by heat transfer or thermal radiation, heat transfer irreversibility (HTI), $\left(S_{th}\right)$; the fourth is caused by micro-rotation $\left(S_{rot}\right)$; and the fifth and sixth terms are caused by mass transfer $\left(S_{dif}\right)$. Therefore, the volumetric rate of local entropy generation is obtained as a linear combination of $\left(S_{th}\right),\left(S_{dis}\right),\left(S_{rot}\right)$, such that

(34) $$\begin{array}{cc} & S_{gen}^{\prime \prime \prime }=\left(S_{th}\right)+\left(S_{dis}\right)+\left(S_{rot}\right). \end{array}$$

It is suitable to write the entropy generation number ($N_{G}$) as a ratio between ($S_{gen}^{\prime \prime \prime }$) and a rate of entropy generation ($S_{0}^{\prime \prime \prime }$) where $S_{0}^{\prime \prime \prime }$ is given as

(35) $$\begin{array}{c} S_{0}^{\prime \prime \prime }=\frac{k_{f}{\left(T_{w}-T_{\infty }\right)}^{2}}{T_{\infty }^{2}{\left(x+b\right)}^{2}}. \end{array}$$

The characteristic entropy generation rate $S_{0}^{\prime \prime \prime }$ demonstrates the optimal entropy generation at which the thermodynamic performance of a system is optimized. Finding $S_{0}^{\prime \prime \prime }$ requires solving an optimization problem which is constrained by the irreversible operations of the system. The physical characteristics of the system is varied until a minimum entropy generation is found.

The entropy generation number $N_{G}$ can be obtained as

(36) $$\begin{array}{cc} & N_{G}=\frac{S_{gen}^{\prime \prime \prime }}{S_{0}^{\prime \prime \prime }}=Re\theta^{\prime^{2}}+\frac{ReBr}{\Omega }\left(\left(1+K\right)f^{{\prime \prime }^{2}}+\left(1+K/2\right)h^{\prime^{2}}\right), \end{array}$$

where χ is the concentration difference, and Br and Ω are the Brinkman number and the temperature difference, respectively. These can be expression as

(37) $$\begin{array}{c} Br=\frac{\mu^{2}u_{w}^{2}\left(x\right)}{k_{f}\Delta T},\;\Omega =\frac{\Delta T}{T_{\infty }}=\frac{T_{w}-T_{\infty }}{T_{\infty }}, \end{array}$$

where *R* is the ideal gas. In Equation (36), there are five irreversibility sources that contribute to the entropy generation number, hence $N_{G}$ may be re-written as $N_{G}=S_{th}+S_{dis}+S_{rot}$, where

(38) $$\begin{array}{c} S_{th}=Re\xi^{-1}\left(1+Nr\right)\theta^{\prime^{2}},\;S_{dis}=\frac{ReBr\xi^{-1}\left(1+K\right)f^{{\prime \prime }^{2}}}{\Omega },\\ S_{rot}=\frac{ReBr\xi^{-2}\left(1+K/2\right)h^{\prime^{2}}}{\Omega }. \end{array}$$

The fraction of irreversibility from each source can be obtained by dividing the irreversibility source by the total irreversibility leading to non-dimensional parameters, such as

(39) $$\begin{array}{c} \gamma_{th}=\frac{S_{th}}{N_{G}},\;\gamma_{dis}=\frac{S_{dis}}{N_{G}},\;\gamma_{rot}=\frac{S_{rot}}{N_{G}}, \end{array}$$

where $\gamma_{th}$ is the fraction of irreversibility due to thermal diffusion, $\gamma_{dis}$ is the fraction of irreversibility due to viscous dissipation, and $\gamma_{rot}$ is the fraction of irreversibility due to micro-rotation.

## 5. Results and Discussion

**Figure 2:**
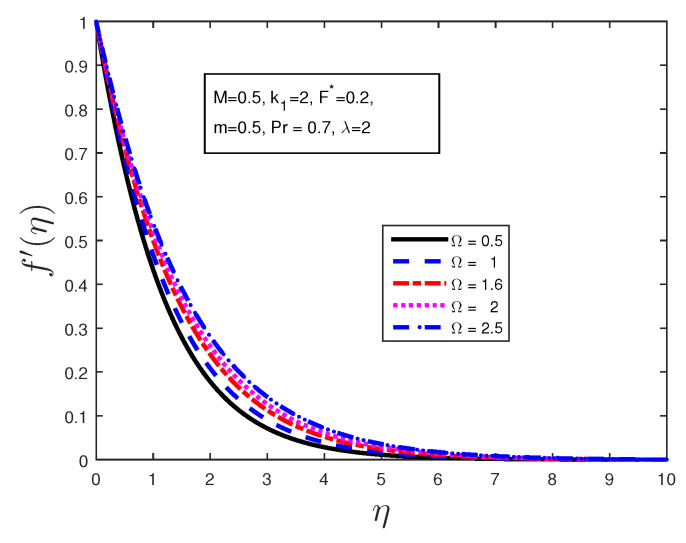
Effect of material parameter Ω on the velocity profile.

**Figure 3:**
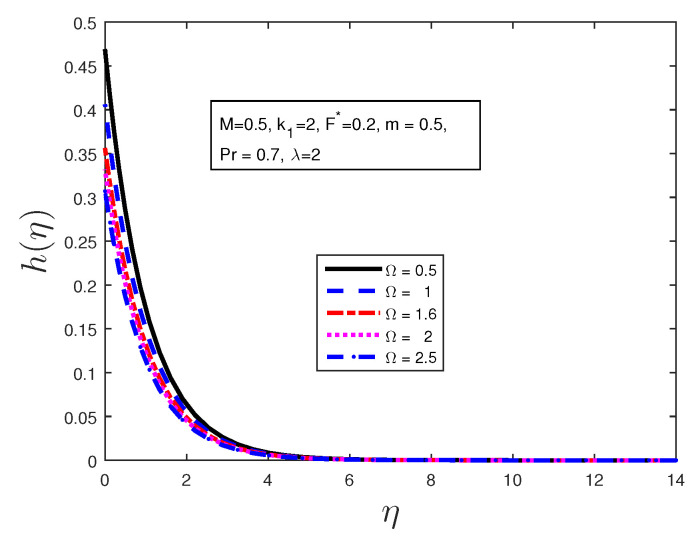
Effect of material parameter Ω on the angular velocity profile.

**Figure 4:**
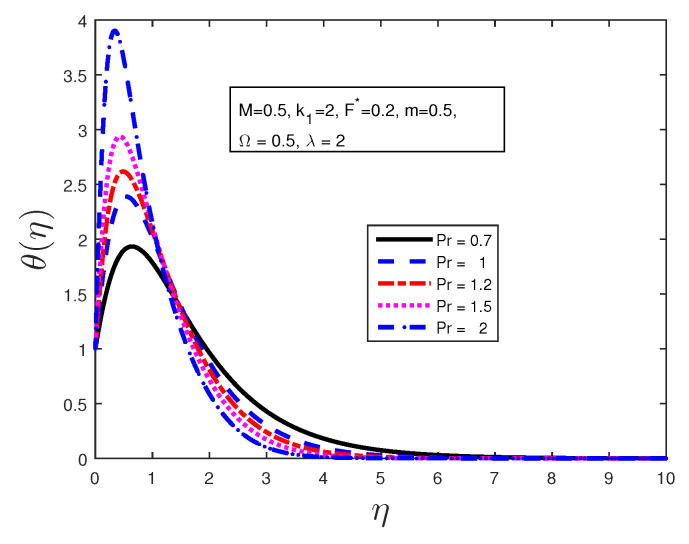
Effect of Prandtl number Pr on the temperature profile.

We investigated the effect of certain parameters on the flow in order to gain a better understanding of the flow dynamics. The results are depicted graphically in the figures below. Table 1 shows the numerical results for the Nusselt number and a comparison is made with previously published work; they are found to be in good agreement. Figure 2 is plotted to discuss the behavior of the velocity profiles for different values of material parameters Ω. It is clear from this figure that an increase in the value of the material parameter leads to an increase in the velocity profile due to the effect of the micropolar fluid. Figure 3 shows the behavior of the angular velocity profiles for different values of material parameters Ω. It can be clearly noted from Figure 3 that the angular velocity decreases when 0≤η≤4, and becomes constant far from the stretching sheet. Figure 4 states the variation of the temperature profiles for various values of Prandtl number Pr. From this figure, it is seen that the temperature decreases with the increasing values of Prandtl number Pr in the boundary layer when η≥1. From this plot, it is evident that the temperature in the boundary layer falls very quickly for large values of Prandtl number; this is because the thickness of the boundary layer decreases with the increase in the value of the Prandtl number. When 0≤η≤1, the temperature profile overshoots for higher values of Prandtl number.

**Figure 5:**
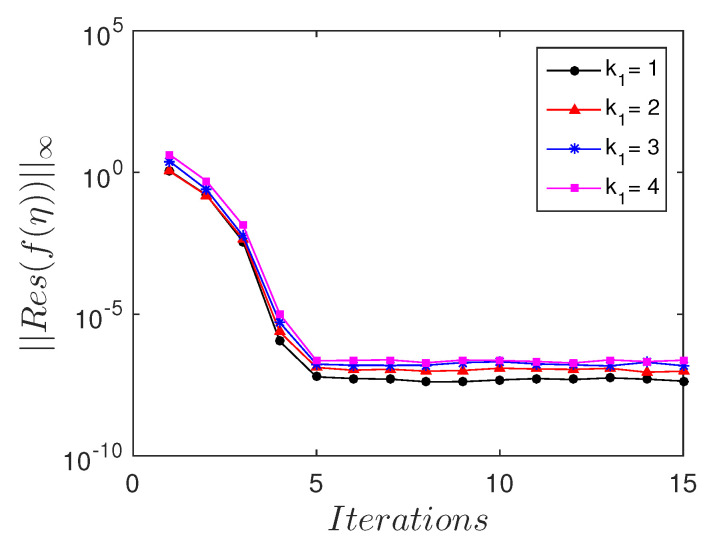
Residual error ${\left||Res\left(f\left(\eta \right)\right)|\right|}_{\infty }$ against the number of iterations, for different values of the porous parameter $k_{1}$.

**Figure 6:**
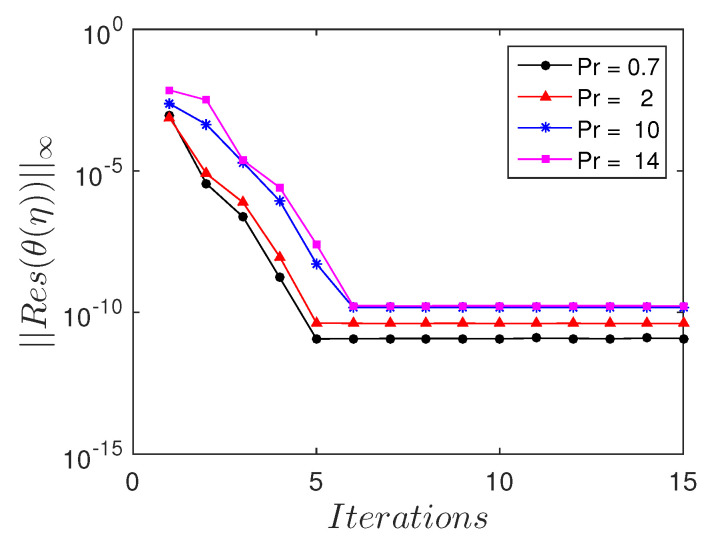
Residual error ${\left||Res\left(f\left(\eta \right)\right)|\right|}_{\infty }$ against the number of iterations, for different values of Prandtl number Pr.

The residual error measures the extent to which the numerical solution approximates the true solution. To gain further understanding of the accuracy of the spectral quasi-linearization method, we calculated the residual errors, as shown in Figure 5 and Figure 6. These are calculated for various values of the porous parameter $k_{1}$ and Prandtl number Pr. In most instances, all the solutions converged with an absolute residual error $\left||\mathrm{Res}|\right|\approx 10^{-10}$ after the third iteration. Accurate solutions were achieved with the least number of iterations.

**Figure 7:**
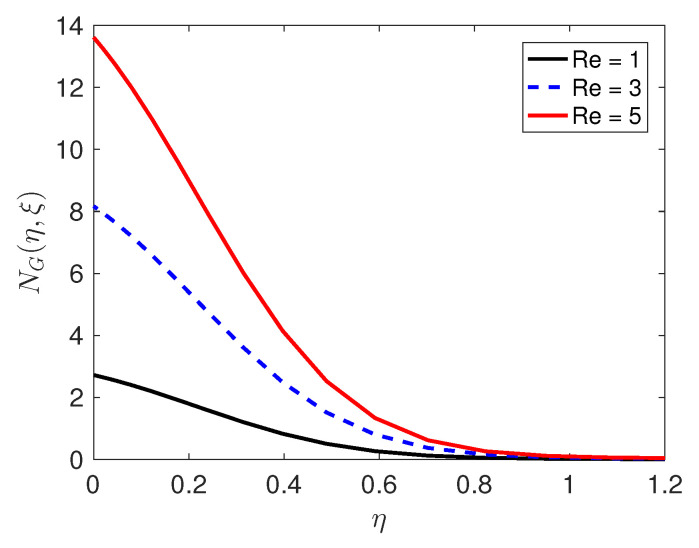
Effect of the Reynolds number Re on the entropy generation profiles.

**Figure 8:**
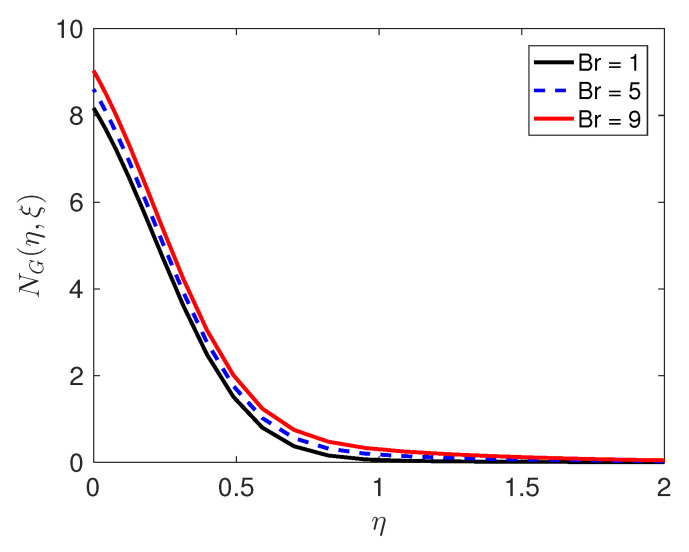
Effect of the Brinkman number Br on the entropy generation profiles.

Entropy generation, which is an important attribute of the flow, is discussed below. The entropy generation is influenced by the quantum and changes in the physical characteristics of the fluid and the porous medium, as can be seen in Equation (36). We consider how physical parameters such as the Reynolds number and the Brinkman number Br impact on entropy generation; the results of this are presented in Figure 7 and Figure 8, respectively. The Reynolds number has a positive impact on entropy generation. The importance of viscous dissipation and fluid conduction are determined by the Brinkman number Br. With increasing Br, viscous dissipation produces more heat which is manifested in the graphs of the temperature profiles. As Brinkman number increases, entropy generation increases. Because entropy generation is responsible for the irreversibility, and our analysis has shown that in that neighborhood of the sheet, the entropy generation is substantially higher in comparison with the other regions, it can be concluded that the sheet is a strong source of irreversibility and thermodynamic imperfections.

## 6. Conclusions

This paper considers the steady and incompressible flow phenomena of a micropolar fluid in which a shrinking or stretching sheet is considered. The effects of radiation on the flow were also taken into account. The spectral quasi-linearization method was used to numerically solve the coupled system of partial differential equations and the results were presented graphically and analyzed. From the discussion above, we can conclude that:

1. There is convergence after a certain number of iterations, demonstrating that the spectral quasi-linearization method is robust and very efficient computationally;
2. The entropy generation increases with increasing Reynolds and Brinkman numbers.

## Figures and Tables

**Table 1:** Comparison of values of $Nu/{Re_{x}}^{\frac{1}{2}}$.

Ω	*m*	Pr	λ	Ref. [5]	Present Results
				HAM Method	Spectral Quasi-Linearization Method (SQLM)
0	−0.2	0.72	1	0.846583	0.845874
0	−0.2	1	1	1.018910	1.018823
0	−0.2	3	1	1.856360	1.855748
0	−0.2	0.72	0	0.382401	0.383021
